# Establishment of tentative epidemiological cutoff values of faropenem against Escherichia coli, Klebsiella pneumoniae, Staphylococcus aureus, Haemophilus influenzae, and Streptococcus pneumoniae

**DOI:** 10.3389/fcimb.2026.1757254

**Published:** 2026-04-22

**Authors:** Shi Wu, Yang Yang, Dandan Yin, Feng Xue, Yun Li, Wei Kang, Qiwen Yang, Jie Lin, Yunsong Yu, Demei Zhu, Yan Guo, Fupin Hu

**Affiliations:** 1 Institute of Antibiotics, Huashan Hospital, Fudan University, and Key Laboratory of Clinical Pharmacology of Antibiotics, Shanghai, China; 2 Department of Laboratory Medicine, State Key Laboratory of Complex Severe and Rare Diseases, Peking Union Medical College Hospital, Chinese Academy of Medical Science and Peking Union Medical College, Beijing, China; 3 Institute of Clinical Pharmacology, Peking University First Hospital, Beijing, China; 4 Department of Laboratory Medicine, Sir Run Run Shaw Hospital, School of Medicine, Zhejiang University, Hangzhou, Zhejiang, China

**Keywords:** antimicrobial susceptibility testing, epidemiological cut-off values, Escherichia coli, faropenem, Haemophilus influenzae, Klebsiella pneumoniae, Staphylococcus aureus, Streptococcus pneumoniae

## Abstract

**Objective:**

This study aimed to establish tentative epidemiological cutoff values (TECOFFs) for faropenem against *Escherichia coli*, *Klebsiella pneumoniae*, *Staphylococcus aureus*, *Haemophilus influenzae*, and *Streptococcus pneumoniae* based on minimum inhibitory concentration (MIC) distributions.

**Methods:**

A total of 2,032 bacterial isolates were collected from Peking University First Hospital, Peking Union Medical College Hospital, Huashan Hospital affiliated with Fudan University, and Sir Run Run Shaw Hospital affiliated with Zhejiang University School of Medicine between 2019 and 2021. The susceptibility of these bacteria to faropenem was determined using the broth microdilution and disk diffusion methods in the clinical microbiology laboratories of these four hospitals. To establish wild-type TECOFFs for faropenem, isolates susceptible to ertapenem for *E. coli* and *K. pneumoniae*, those susceptible to oxacillin for *S. aureus*, and those with unscreened susceptibility for *H. influenzae* and *S. pneumoniae* were regarded as wild-type strains.

**Results:**

The MIC TECOFF for faropenem against *E. coli* was determined to be 2 mg/L, with MIC ≤2 mg/L indicating wild-type strains and MIC ≥4 mg/L indicating non-wild-type strains. The corresponding inhibition zone diameter TECOFF for *E. coli* was 19 mm, where an inhibition zone diameter ≤18 mm indicates non-wild-type strains and ≥19 mm indicates wild-type strains. For *K. pneumoniae*, the MIC TECOFF was also 2 mg/L, with similar cutoffs established for the inhibition zones. The MIC TECOFF for faropenem against *S. aureus* was 0.25 mg/L, for *H. influenzae* was 1 mg/L, and for *S. pneumoniae* was 2 mg/L. The corresponding inhibition zone diameter ECOFF for faropenem against *S. aureus* was 31 mm, for *H. influenzae* was 14 mm, and for *S. pneumoniae* was 33 mm.

**Conclusion:**

The TECOFFs for faropenem against the tested bacteria were established. These data are useful for clinical microbiologists and clinicians in the interpretation of the results of faropenem susceptibility testing.

## Introduction

Faropenem is a β-lactam antibiotic developed by Suntory in Japan, classified as a penem derivative and one of the few orally administered drugs in the penem class (Gettig et al., 2008). In November 2006, faropenem was approved by the China Food and Drug Administration (CFDA) for the treatment of infections caused by sensitive strains of *Escherichia coli*, *Klebsiella pneumoniae*, *Haemophilus influenzae*, *Staphylococcus aureus*, *Streptococcus pneumoniae*, and *Bacteroides* species (The State Food and Drug Administration approves the launch of faropenem). Faropenem has the advantage of being well absorbed orally, eliminating the need for injection, thereby improving patient compliance. Studies indicate that while other marketed penem antibiotics such as meropenem (Peng et al., 2025) exhibit varying degrees of nephrotoxicity, preclinical studies of faropenem sodium have shown no nephrotoxicity in dogs even at doses up to 2,000 mg/kg for 26 consecutive weeks (Faqi et al., 2010). *In vitro* susceptibility testing revealed that faropenem has high antibacterial activity against pathogens responsible for both community- and hospital-acquired infections, including extended-spectrum β-lactamase (ESBL)-producing Enterobacterales, methicillin-susceptible *S. aureus* (MSSA), and penicillin-susceptible *S. pneumoniae* (Turnidge et al., 2006).

Antimicrobial susceptibility testing provides essential foundational data for the rational use of antibiotics, which helps clinicians to select the most appropriate antimicrobial therapy for specific pathogens. However, neither the Clinical and Laboratory Standards Institute (CLSI) (Performance standards for antimicrobial susceptibility testing. CLSI supplement M100. 31th ed, 2021) nor the European Committee on Antimicrobial Susceptibility Testing (EUCAST) (European Committee on Antibiotic Susceptibility Testing) has yet established clinical breakpoints for faropenem against any pathogens, including epidemiological cutoff values (ECOFFs). The aim of this study was to establish tentative epidemiological cutoff values (TECOFFs) for faropenem against *E. coli*, *K. pneumoniae*, *S. aureus*, *H. influenzae*, and *S. pneumoniae* based on the distribution of inhibition zone diameters and minimum inhibitory concentrations (MICs).

## Materials and methods

### Study design

The study design is primarily based on the EUCAST guidelines for the establishment of TECOFFs (European Committee on Antibiotic Susceptibility Testing) for antimicrobial agents. In addition, it takes into account the “Technical Guidelines for Research on Antimicrobial Susceptibility Testing Breakpoints” issued by the Center for Drug Evaluation (CDE) of the National Medical Products Administration (NMPA) in 2018 (The State Food and Drug Administration), which include rules for antimicrobial susceptibility testing methodologies, wild-type strain definitions, laboratory selection, and epidemiological cutoff value determination.

### Clinical isolates

From 2019 to 2021, a total of 2,032 non-duplicate clinical isolates were collected from the laboratories of four hospitals in China. These included *E. coli* (*n* = 407), *K. pneumoniae* (*n* = 399), *S. aureus* (*n* = 404), *H. influenzae* (*n* = 411), and *S. pneumoniae* (*n* = 411). Quality control strains for susceptibility testing included *S. aureus* ATCC 29213 and ATCC 25923, *E. coli* ATCC 25922, *H. influenzae* ATCC 49766, and *S. pneumoniae* ATCC 49619.

### Antimicrobial susceptibility testing

The susceptibility of faropenem was determined in the four laboratories using the broth microdilution and disk diffusion methods according to the CLSI M100 31st edition guidelines. To ensure consistency and reliability, materials such as broth microdilution plates, antimicrobial susceptibility disks, and cation-adjusted Mueller–Hinton broth (CAMHB) were uniformly provided by the central laboratory (Clinical Microbiology Laboratory of the Antibiotic Research Institute, Huashan Hospital, Fudan University). Faropenem standard pure powder (93.2% potency, lot 190606) was provided by Hengtong Pharmaceutical Co. (Shaanxi, China). Faropenem susceptibility disks (5 μg/disk, lot 07103) were purchased from Nissui Pharmaceutical Co., Ltd. (Tokyo, Japan). CAMHB (BD, Franklin Lakes, NJ, USA) and Mueller–Hinton agar (MHA; Oxoid, Basingstoke, UK) were used for the broth microdilution and disk diffusion methods. For fastidious organisms, MHA was supplemented with defibrinated sheep blood or horse blood and SR0158E supplement (Oxoid, Basingstoke, UK) for *H. influenzae* following standard formulations. All media were prepared following the manufacturers’ instructions. Faropenem was dissolved and diluted in sterile distilled water with a concentration range of 256–0.008 mg/L (16 concentrations). Strain identification was performed using the VITEK^®^ MS microbial identification system (bioMérieux, Marcy-l’Étoile, France).

### Participating laboratories

The study was conducted by four laboratories: the Clinical Microbiology Laboratory of the Antibiotic Research Institute, Huashan Hospital Fudan University (central laboratory); the Clinical Microbiology Laboratory of Peking Union Medical College Hospital; the Clinical Pharmacology Institute of Peking University First Hospital; and the Clinical Microbiology Laboratory of Sir Run Run Shaw Hospital, Zhejiang University School of Medicine. The strain distribution is listed in **Table 1**.

**Table 1:** Strain distribution from the four laboratories in this study.

Laboratory	*N*	*Escherichia coli*	*Haemophilus influenzae*	*Klebsiella pneumoniae*	*Staphylococcus aureus*	*Staphylococcus pneumoniae*
The Clinical Microbiology Laboratory of the Antibiotic Research Institute, Huashan Hospital, Fudan University	537	108	114	100	104	111
The Clinical Pharmacology Institute of Peking University First Hospital	500	100	100	100	100	100
The Clinical Microbiology Laboratory of Peking Union Medical College Hospital	495	99	97	99	100	100
The Clinical Microbiology Laboratory of Sir Run Run Shaw Hospital, Zhejiang University School of Medicine	500	100	100	100	100	100
Total	2,032	407	411	399	404	411

### Definition of wild-type strains

Based on the EUCAST definitions, wild-type strains are those that do not carry acquired or mutational resistance mechanisms to faropenem (or similar antibiotics) (European Committee on Antibiotic Susceptibility Testing). In this study, *E. coli* and *K. pneumoniae* strains susceptible to ertapenem, *S. aureus* strains susceptible to oxacillin, and *H. influenzae* and *S. pneumoniae* strains without prescreened susceptibility were defined as wild-type strains. These were used to analyze the TECOFFs for faropenem against clinical isolates.

### Data analysis

Data were processed and analyzed using WHONET 5.6 software to determine parameters such as MIC range, MIC distributions, and inhibition zone diameter distributions for faropenem against the bacterial clinical isolates. The ECOFFinder software was used to analyze the MIC TECOFFs for faropenem against *E. coli*, *K. pneumoniae*, *S. aureus*, *H. influenzae*, and *S. pneumoniae*. The tool automatic_NRI-zone_2019 (http://www.bioscand.se/nri/) was used to calculate the tentative inhibition zone diameter TECOFFs for these bacterial species (The State Food and Drug Administration).

### Ethical approval

This study was approved by the Ethics Committee of Huashan Hospital, Fudan University (approval no. 2020, clinical review no. 2018-453). The strains used in the study were directly selected from isolates previously approved by ethics committees (no. KY2018-453), and all strains underwent de-identification processing (e.g., removal of patient privacy information).

## Results

### Escherichia coli

The MIC range of faropenem for 407 strains of *E. coli* was 0.125–16 mg/L, with MIC_50_ and MIC_90_ values of 0.5 and 2 mg/L, respectively. The inhibition zone diameter for *E. coli* ranged from 9 to 32 mm. Statistical analysis showed that the TECOFF for faropenem against *E. coli* was 2 mg/L. Strains with MIC ≤2 mg/L were categorized as wild type, while those with MIC ≥4 mg/L were classified as non-wild type. The inhibition zone TECOFF for faropenem (5 µg) was 19 mm: those with zone diameters ≤18 mm are non-wild type, and those with ≥19 mm are wild type. (**Tables 2**–**4** and **Supplementary Data**).

**Table 2:** Antimicrobial activity of faropenem against selected bacterial species determined by the broth microdilution method.

Organism	MIC range	MIC_50_	MIC_90_	MIC mode	No. of isolates with MIC values (μg/ml)												
					≤0.008	0.015	0.03	0.06	0.125	0.25	0.5	1	2	4	8	16	32
*Escherichia coli* (*n* = 407)	0.125–16	0.5	2	0.5					3	51	208	102	23	13	6	1	
*Klebsiella pneumoniae* (*n* = 399)	0.125–32	0.5	2	0.5					1	58	202	65	34	17	12	8	2
*Staphylococcus aureus* (*n* = 404)	0.03–1	0.125	0.25	0.125			1	20	337	35	10	1					
*Haemophilus influenzae* (*n* = 411)	0.008–32	0.25	2	0.25	37	50	24	38	33	64	61	41	38	18	6		1
*Staphylococcus pneumoniae* (*n* = 411)	0.008–2	0.25	1	0.5	68	66	20	8	25	59	102	52	11				

MIC, minimum inhibitory concentration.

**Table 3:** Antimicrobial activity of faropenem against *Escherichia coli*, *Klebsiella pneumoniae*, and *Haemophilus influenzae* determined using the disk diffusion method.

Organism	No. of isolates with inhibition zone diameter (mm)																										
	6	7	8	9	10	11	12	13	14	15	16	17	18	19	20	21	22	23	24	25	26	27	28	29	30	31	≥32
*E. coli* (*n* = 407)				1	2		2		4	1	5	1	9	11	36	28	81	83	79	31	18	7	1	1		3	3
*K. pneumoniae* (*n* = 399)	6	1	3	2	1			4	6		7	9	11	20	18	45	82	81	54	41	7		1				
*H. influenzae* (*n* = 411)	6	1			2	1	4	6	10	11	15	27	24	33	32	24	32	26	29	29	25	25	21	7	9	5	7

**Table 4:** Tentative epidemiological cutoff values (TECOFF) of faropenem for selected bacterial species.

Organism	MIC of TECOFF (μg/ml)		Inhibition zone diameter of TECOFF (mm)	
	WT	NWT	WT	NWT
*Escherichia coli* (*n* = 407)	≤2	≥4	≥18	≤17
*Klebsiella pneumoniae* (*n* = 399)	≤2	≥4	≥18	≤17
*Staphylococcus aureus* (*n* = 404)	≤0.25	≥0.5	≥31	≤30
*Haemophilus influenzae* (*n* = 411)	≤1	≥2	≥14	≤13
*Staphylococcus pneumoniae* (*n* = 411)	≤2	≥4	≥33	≤32

MIC, minimum inhibitory concentration; WT, wild type; WT, non-wild type.

### Klebsiella pneumoniae

The MIC range of faropenem for 399 strains of *K. pneumoniae* was 0.125–32 mg/L, with MIC_50_ and MIC_90_ values of 0.5 and 2 mg/L, respectively. The inhibition zone diameter for *K. pneumoniae* ranged from 6 to 28 mm. Statistical analysis showed that the TECOFF for faropenem against *K. pneumoniae* was 2 mg/L. Strains with MIC ≤2 mg/L were classified as wild type, while those with MIC ≥4 mg/L were categorized as non-wild type. The inhibition zone TECOFF for faropenem (5 µg) was 19 mm: those with zone diameters ≤18 mm are non-wild type, and those with ≥19 mm are wild type. (**Tables 2**–**4** and **Supplementary Data**).

### Staphylococcus aureus

The MIC range of faropenem for 404 strains of *S. aureus* was 0.03–1 mg/L, with MIC_50_ and MIC_90_ values of 0.125 and 0.25 mg/L, respectively. The inhibition zone diameter for *S. aureus* ranged from 25 to 47 mm. The inhibition zone TECOFF for faropenem (5 µg) was 31 mm: those with zone diameters ≤30 mm are non-wild type, and those with ≥31 mm are wild type. (**Tables 2**, **5**, **4** and **Supplementary Data**).

**Table 5:** Antimicrobial activity of faropenem against *Staphylococcus aureus* and *Staphylococcus pneumoniae* determined with the disk diffusion method.

Organism	No. of isolates with inhibition zone diameter (mm)																										
	21	22	23	24	25	26	27	28	29	30	31	32	33	34	35	36	37	38	39	40	41	42	43	44	45	46	≥47
*S. aureus* (*n* = 404)					3	1		2				1	7	26	85	92	100	42	24	9	5	1	4				2
*S. pneumoniae* (*n* = 411)	6	26	10	27	24	35	31	24	21	23	8	11	22	13	11	14	16	13	9	14	11	7	14	3	10	2	6

### Haemophilus influenzae

The MIC range of faropenem for 411 strains of *H. influenzae* was 0.008–32 mg/L, with MIC_50_ and MIC_90_ values of 0.25 and 2 mg/L, respectively. The MIC TECOFF for faropenem against *H. influenzae* was 1 mg/L. Strains with MIC ≤1 mg/L were classified as wild-type, while those with MIC ≥2 mg/L were categorized as non-wild type. The inhibition zone diameter for *H. influenzae* ranged from 6 to ≥32 mm. The inhibition zone TECOFF for faropenem (5 µg) was 14 mm: those with zone diameters ≤13 mm are non-wild type, and those with ≥14 mm are wild type. (**Tables 2**–**4** and **Supplementary Data**).

### Streptococcus pneumoniae

The MIC range of faropenem for 411 strains of *S. pneumoniae* was ≤0.008–2 mg/L, with MIC_50_ and MIC_90_ values of 0.25 and 1 mg/L, respectively. The MIC TECOFF for faropenem against *S. pneumoniae* was 2 mg/L. Strains with MIC ≤2 mg/L were categorized as wild type, while those with MIC ≥4 mg/L categorized as non-wild type. The inhibition zone diameter for *S. pneumoniae* ranged from 21 to ≥47 mm. The inhibition zone TECOFF for faropenem (5 µg) was 33 mm: those with zone diameters ≤32 mm are non-wild type, and those with ≥33 mm are wild type. (**Tables 2**, **5**, **4** and **Supplementary Data**).

## Discussion

Clinically common pathogens such as *E. coli*, *K. pneumoniae*, *S. aureus*, *H. influenzae*, and *S. pneumoniae* are important bacterial pathogens in healthcare settings (Hu et al., [[NoYear]]; Di Pilato et al., 2024; Hsueh et al., 2024). These pathogens cause an increasing number of infections in clinical practice and are also among the most common causative agents of hospital-acquired bloodstream infections. In addition, pathogens such as Gram-negative bacilli often exhibit increasing antibiotic resistance, particularly carbapenem-resistant Gram-negative bacilli (Abdulqawi et al., 2024). The increasing prevalence of antibiotic-resistant bacteria is a major challenge for antimicrobial therapy (Oliver et al., 2024). The development of new antimicrobial agents is a critical strategy to addressing the problem of infections caused by drug-resistant bacteria. Faropenem is a significant achievement in the search for new drugs to combat resistant bacterial infections. As a novel β-lactam antibiotic, faropenem belongs to the penem class of antibiotics. Its mechanism of action is the blockage of bacterial cell wall synthesis by binding tightly to the penicillin-binding proteins (PBPs), thereby exerting bactericidal effects. Faropenem has demonstrated excellent *in vitro* activity against target pathogens (Nayak et al., 2022), outperforming existing oral cephalosporins in terms of antimicrobial activity, and has emerged as the preferred treatment for severe infections in hospital settings (Gandra et al., 2022).

In this study, we determined the MICs and inhibition zone diameters of faropenem for selected bacteria using the broth microdilution and disk diffusion methods, respectively. With the ECOFFinder software (European Committee on Antibiotic Susceptibility Testing) and visual inspection, we estimated the TECOFFs for different bacterial species through statistical analysis. The TECOFF represents the highest MIC for wild-type strains that lack phenotypically detectable acquired resistance mechanisms (upper limit of the wild-type MIC distribution). The TECOFF can be used to classify isolates as either wild type or non-wild type. We defined wild-type strains as follows: *E. coli* and *K. pneumoniae* strains that are susceptible to ertapenem; *S. aureus* strains that are susceptible to oxacillin; and *H. influenzae* and *S. pneumoniae* strains without prescreened susceptibility data. Subsequently, we calculated the MIC distributions, MIC ranges, MIC_50_ MIC_90_ values, and the corresponding TECOFF for faropenem (see **Table 4**). Our data and calculations are consistent with the standards defined in the EUCAST guidelines for establishing wild-type MIC distributions and setting the TECOFF values.

By comparing the MIC ranges obtained in this study with findings from multiple global epidemiological surveys, we found a high degree of geographical consistency overall. For *E. coli*, a systematic review summarizing multiple *in vitro* studies reported an MIC range of 0.12–2 mg/L (Gandra et al., 2022). The MIC distribution for *K. pneumoniae* showed greater variability within the species, with the MIC_90_ values typically ranging from 1 to 4 mg/L (Gandra et al., 2022). In contrast, multiple studies have reported highly consistent MIC_90_ values concentrated between 0.12 and 0.25 mg/L (Woodcock et al., 1997), which was attributed to a high affinity for PBPs (Sayood et al., 2025). For *H. influenzae*, the reported MIC_90_ values ranged from 0.5 to 1 mg/L, unaffected by the β-lactamase production (Walsh et al., 2003). Notably, the *H. influenzae* isolate with an MIC of 32 mg/L in our study is unusual, reflecting potential resistance mechanisms such as PBP mutations or efflux pump overexpression. For *S. pneumoniae*, the globally reported MIC_90_ is typically between 0.5 and 1 mg/L (Critchley et al., 2002). The MIC_90_ in this study was 1 mg/L, which is at the upper limit.

According to the EUCAST-defined TECOFF MIC distributions for faropenem, all of the strains in this study were wild type for faropenem susceptibility. It is important to note that non-wild-type isolates may have reduced susceptibility to antibiotics due to acquired resistance mechanisms (Fujino et al., 2017). Among the Enterobacterales, faropenem resistance mutations are primarily associated with non-synonymous changes in the *ompC* gene and single nucleotide polymorphisms (SNPs) in the *envZ* gene. In addition, there is cross-resistance between faropenem and other commercially available carbapenem antibiotics (Gandra et al., 2020).

Although this study developed TECOFFs for faropenem against *E. coli*, *K. pneumoniae*, *S. aureus*, *H. influenzae*, and *S. pneumoniae*, which inform the conduct of essential antimicrobial susceptibility testing for faropenem, the development of TECOFF for antimicrobial agents is only the first step. However, TECOFFs can provide a scientific reference for the subsequent development of pharmacokinetics/pharmacodynamics (PK/PD) and clinical breakpoints. As only four laboratories participated in this study and the sample size for each bacterium was not large, how long the TECOFFs developed in this study can be applied for needs to be confirmed by more subsequent studies, including participation of more laboratories, larger sample sizes, and PK/PD studies. Ultimately, of course, clinical evaluation will be required to determine whether the ECOFFs need to be revised.

## Data Availability

The original contributions presented in the study are included in the article/**Supplementary Material**. Further inquiries can be directed to the corresponding authors.
